# Annual acknowledgement of manuscript reviewers 2014

**DOI:** 10.1186/s12982-015-0024-9

**Published:** 2015-04-19

**Authors:** Clarence Tam

**Affiliations:** Emerging Themes in Epidemiology, London, UK

## Abstract

The editor of *Emerging Themes in Epidemiology* would like to thank all our reviewers who have contributed to the journal in Volume 11 (2014).

**Cornelia Adlhoch**

Sweden

**Neal Alexander**

United Kingdom

**Wladimir Alonso**

United States of America

**Ben Armstrong**

United Kingdom

**Kari Auranen**

Finland

**Julie Balen**

United Kingdom

**Larry Beeson**

United States of America

**M. Hanna Bos**

Netherlands

**Laura Brown**

United States of America

**Anne Buchanan**

United States of America

**Nicole Carnegie**

United States of America

**Yan Che**

China

**Cynthia Chee**

Singapore

**Stuart Clarke**

United Kingdom

**Elizabeth Costenbader**

United States of America

**Douglas Curran-Everett**

United States of America

**Olaf Dekkers**

Netherlands

**Frank Dudbridge**

United Kingdom

**Conrad Fernandez**

Canada

**Lea Fortunato**

United Kingdom

**William Gray**

United Kingdom

**Diane Gross**

United States of America

**Andrew Hayward**

United Kingdom

**Donald Henderson**

United States of America

**William Johnson**

United Kingdom

**Amalia Karahalios**

Australia

**Mishal Khan**

Pakistan

**Carl Koppeschaar**

Netherlands

**Marleen Kraaij - Dirkzwager**

Netherlands

**Karl Kuschner**

United States of America

**Vincenzo Lagani**

Greece

**Heidi Larson**

United Kingdom

**Andrew Lover**

United States of America

**Joelle Mak**

United Kingdom

**Samuel Manda**

South Africa

**Punam Mangtani**

United Kingdom

**Patricia Mechael**

United States of America

**Ziad Memish**

Saudi Arabia

**Daniela Paolotti**

Italy

**Daniel Payne**

United States of America

**Neil Pearce**

United Kingdom

**Wirichada Pongtavornpinyo**

Thailand

**Charles Poole**

United States of America

**Naghma Rehan**

Pakistan

**Cristina Rodriguez Sanchez**

Spain

**Daniel Roth**

Canada

**Jalil Safaei**

Canada

**M A Yushuf Sharker**

Bangladesh

**Jet Smit**

Netherlands

**Eva Steliarova-Foucher**

France

**Jan Stochl**

United Kingdom

**Andrew Tai**

Australia

**Clare Tanton**

United Kingdom

**Laurie Tomlinson**

United Kingdom

**Pierre Traissac**

France

**Arianne B van Gageldonk-Lafeber**

Netherlands

**Sukumar Vellakkal**

United Kingdom

**Damja Vukcevic**

Australia

**Clarice Waters**

Singapore

**Roger Watson**

United Kingdom

**Elizabeth Webb**

United Kingdom

**Douglas Weed**

United States of America

**Helen Weiss**

United Kingdom

**Laura Woods**

United Kingdom

**Chen-Yi Wu**

Taiwan

**Yan Zheng**

United States of America

